# Nocardia Brain Abscess in a Patient With Sarcoidosis

**DOI:** 10.7759/cureus.32759

**Published:** 2022-12-20

**Authors:** Fatima Ghazal, Sylvia Botchway, Edgar Naut

**Affiliations:** 1 Internal Medicine, University of Connecticut Health, Farmington, USA; 2 Hospital Medicine, Saint Francis Hospital, Hartford, USA

**Keywords:** central nervous system, cns tumors, brain tumors, infectious mass effect, nocardia brain infection, central nervous system infections (cns), brain abscess, nocardia infection

## Abstract

*Nocardia* species are aerobic, gram-positive, filamentous bacteria. Infection occurs either through inhalation leading to pulmonary symptoms or inoculation presenting with skin findings. Hematogenous dissemination, although uncommon, is possible and can lead to central nervous system involvement. *Nocardia* brain abscess is a rare manifestation that comprises 2% of all brain abscess etiologies, mostly occurring in immunocompromised patients. Establishing a diagnosis is often difficult, especially due to its rare occurrence and the fact that it can mimic other etiologies on brain imaging including necrotic tumors. High mortality rates have been reported due to delays in establishing a diagnosis and a lack of precise treatment guidelines.

## Introduction

*Nocardia* species is mainly an opportunistic organism causing infections in immunocompromised hosts with only one-third of patients reported to be immunocompetent [1]. Localized infections primarily occur in the lung or the skin. Hematogenous spread can also occur causing systemic infections such as central nervous system (CNS) nocardiosis [2]. Cerebral nocardiosis is rare and usually manifests as brain abscesses, although cerebral infiltration, meningitis, ventriculitis, and spinal cord infections have been reported [3]. Nocardial brain abscess accounts for 2% of all cerebral abscesses carrying the highest mortality rate among bacterial abscesses [2,4,5].

Clinical presentations of nocardial brain abscesses are variable. Literature reports a wide range of neurological manifestations, including change in mental status, change in personality, visual deficits, foot drop, lower extremity paresthesia, spasticity, and multiple other motor and sensory deficits. Some others lacked neurological deficits and presented with systemic manifestations such as low-grade fever and weight loss [1,5]. Misdiagnosis is common due to multiple factors such as difficulty in diagnosis, paucity of disease, and variability of clinical presentations [2,5,6]. Imaging is the first step in diagnosis and findings can consist of one or multiple ring-enhancing lesions on computed tomography (CT) or magnetic resonance imaging (MRI) [1,3,7]. These radiological findings raise concern for other conditions, including necrotic neoplasms, infected tumors, pyogenic abscesses, and stroke, causing a delay in diagnosis [3,7]. Definite diagnosis of nocardial brain abscess requires isolation and culture of bacteria from blood, CSF, or abscess aspirates [1,3,5,8].

Optimal treatment for CNS nocardiosis has not been established [1]. Literature reported a combination of regimens that have been used, including antimicrobial therapy, aspiration, craniotomy, or a combination of both medical and surgical management depending on the size and location of the brain abscess and response to treatment [2,5].

## Case presentation

A 69-year-old male patient with a past medical history of paroxysmal atrial fibrillation on apixaban, bipolar disorder, and pulmonary sarcoidosis on long-term steroid therapy with prednisone 10 mg daily presented with a chief complaint of confusion and headache for several weeks. History was obtained from the patient’s wife, who reported the patient lives in a suburban area and enjoys fishing and gardening. He has been having daily headaches that started four weeks prior to the presentation lasting four hours. This was associated with a change in mental status, which manifested as confusion and difficulty completing daily activities. His confusion worsened a week prior to the presentation, prompting the emergency department visit. There was no associated nausea, vomiting, or change in vision. The headaches were temporarily relieved with analgesics. His initial vitals showed a blood pressure of 111/76 mmHg, heart rate of 81, respiratory rate of 16, and temperature of 36.4°C. On physical examination, the patient was somnolent but easily aroused, and oriented to person and place only. His speech was clear and fluent, but he was notably confused. He was not cooperative with a neurologic exam. He had a full passive range of motion in all extremities. Reflexes across all extremities were symmetric and preserved. No abnormal findings were reported on respiratory, cardiac, or abdominal examination.

**Figure 1:**
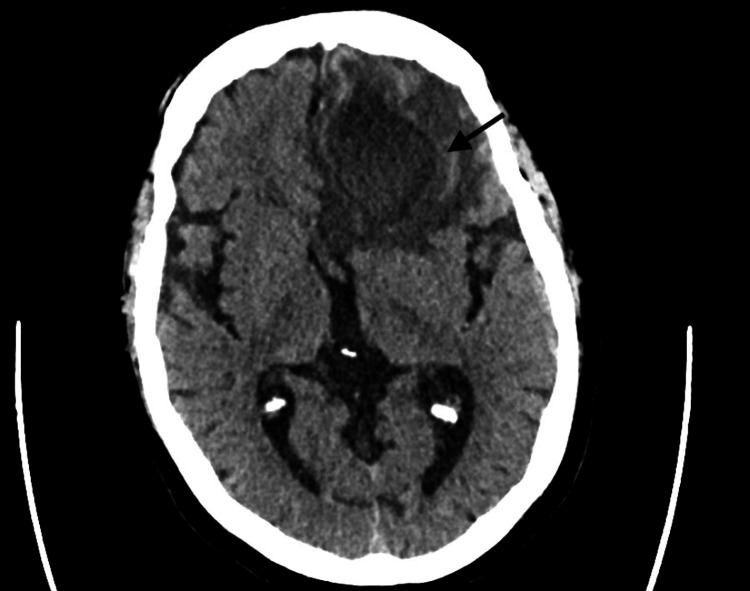
CT of the head without contrast CT of the head without contrast revealed a solitary 3.5 cm mass in the left frontal lobe with vasogenic edema and mass effect upon the anterior horn of the left lateral ventricle.

**Figure 2:**
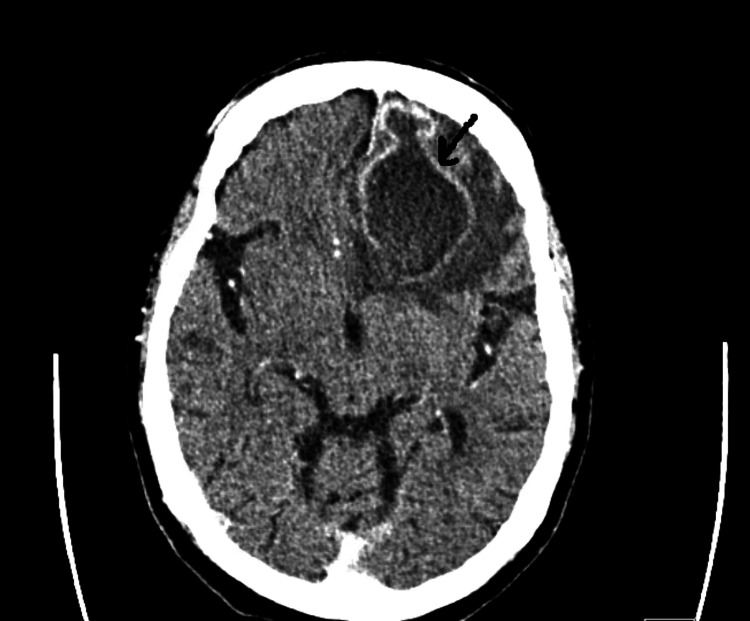
CT of the head with contrast CT of the head with contrast revealed a peripherally enhancing mass in the left frontal lobe.

Lab work on presentation showed a WBC count of 8,100 per microliter (mL), and creatinine of 1.1 milligram/deciliter (mg/dL) with no electrolyte disturbance noted. The patient’s thyroid function test, liver function tests, coagulation panel, electrolytes, and ammonia levels were within normal limits. Ethanol, urine analysis, and urine drug screen were negative. ECG showed normal sinus rhythm. Chest radiography was unremarkable. CT of the head without contrast revealed a solitary 3.5 cm mass in the left frontal lobe with vasogenic edema and mass effect upon the anterior horn of the left lateral ventricle (Figure 1). This has been reported as a peripherally enhancing mass in the left frontal lobe on the CT of the head with contrast (Figure 2). While in the emergency room, the patient had a seizure, which responded to lorazepam 1 milligram (mg).

The patient was started on levetiracetam for seizure prophylaxis and dexamethasone for vasogenic edema. MRI of the brain was not performed because the patient had an inactive pacemaker that was not MRI-compatible. An electroencephalogram was performed with epileptiform discharges noted. A CT of the abdomen and pelvis was negative. CT of the chest revealed calcified lymph nodes consistent with an old granulomatous disease but no concern for active infection. After holding anticoagulation for five days, a left frontal craniotomy was performed during which a 5 cm capsule with pus was noted and the abscess was drained. A follow-up CT of the head was performed and showed successful decompression of the brain abscess. He was started empirically on cefepime, vancomycin, and metronidazole. Azathioprine was started for the management of sarcoidosis in place of prednisone. The fluid culture gram stain grew aerobic, branching gram-positive bacteria in a beaded pattern with concern for *Nocardia*. Cefepime was de-escalated to ceftriaxone, and the patient was started on imipenem and Bactrim in addition to metronidazole and vancomycin. His hospital course was complicated by recurrence of seizure activity with repeat CT of the head revealing a new hemorrhage involving the left frontal lobe at the site of abscess drainage with persistence in midline shift and new periorbital edema. These findings were attributed to the sequelae of the craniotomy. He also developed a syndrome of inappropriate antidiuretic hormone secretion (SIADH) with sodium fluctuating in the range of 125-130 mmol/L. Although seizure activity occurred prior to any sodium derangement, correcting the sodium level was a priority to avoid further triggering factors. Valproic acid was added as a second anti-epileptic agent and the patient completed a course of dexamethasone. The final body fluid culture grew *Nocardia farcinica*, and blood cultures remained negative. After clinical improvement and stabilization of CT head findings, the patient was eventually discharged to a long-term acute care hospital on Bactrim and amikacin to complete a six-week course of antibiotics.

His post-discharge course was complicated by hallucinations, which were attributed to levetiracetam. Symptoms resolved with discontinuation. He also developed pancytopenia attributed to Bactrim, which was replaced with ciprofloxacin for one year. At his one-year follow-up with infectious disease, the patient had no neurological deficits noted.

## Discussion

*Nocardia* is an aerobic, branching gram-positive rod that can affect both immunocompromised and immunocompetent hosts. Primary infections occur mainly in the lungs or the skin through inhalation or direct inoculation, respectively [2]. The Centers for Disease Control and Prevention (CDC) reported an estimated 500-1,000 cases of nocardiosis infection occurring yearly in the United States, with around 60% of these cases associated with immunocompromised patients [9]. Given that the bacteria is found in standing water and soil, farmers and swimmers have a high risk of exposure [9,10]. Systemic infections can occur through the hematogenous spread and are more frequently found in immunocompromised patients such as those with autoimmune diseases, cell-mediated immune defects, malignancies, or those on long-term steroids [1-3]. The patient described above was immunocompromised due to chronic steroid use for sarcoidosis with hobbies that include fishing and gardening, which are considered risk factors for *Nocardia* infection. He presented with a rare manifestation of CNS nocardiosis, which caused a large brain abscess without primary lung or skin manifestations. Nocardial brain abscesses are responsible for 1-2% of all reported bacterial brain abscesses, with more than half of these infections occurring in immunosuppressed patients [11,12]. Literature is limited to case reports and few case series published, although incidence seems to be growing [3]. Sixteen different species of *Nocardia* are known to cause clinical manifestations in humans, with the most commonly reported species in the literature being *N. asteroides*, *N. farcinica*, and *N. cyriacigeorgica* [1,4].

Nocardial brain abscess has a poor prognosis due to the rapid clinical deterioration with a high risk of mortality and morbidity [2,12]. According to Solano-Varela et al., mortality of nocardial CNS infection can be as high as 57% in immunocompromised patients [8]. While the CDC reports a 44% mortality rate in patients with *Nocardia* infection spread to the brain or spine, with the rate increasing to 85% in immunocompromised patients [9]. Symptoms of CNS nocardiosis are variable and non-specific [1]. In the literature, a case was reported where a patient with CNS *Nocardia* presented without any systemic symptoms, the sole complaint was seizures, which is a similar presentation to our patient [5]. He had a rapid decline in his mental status over the course of a few weeks, he had no focal neurological deficits and had a witnessed seizure during his hospitalization. Identifying a brain abscess on CT or MRI imaging and differentiating it from brain tumors is very difficult due to their similar structural findings. This is a major contributing factor that leads to misdiagnosis and delay in treatment [7]. MRI has been found to be superior in distinguishing brain abscesses from neoplasms, although the specificity is low [7]. Our patient was thought to have a brain tumor on initial presentation. An MRI was not feasible due to the presence of an incompatible pacemaker. Further imaging did not isolate a primary tumor. The absence of the classic skin and pulmonary manifestations, the limitations with obtaining further imaging, and his rapid clinical decline were all factors that contributed to the delayed diagnosis. The gold standard for making the diagnosis of a nocardial infection is through isolation of the bacteria in a culture, which in itself can take up to 14-21 days to be finalized. A high level of suspicion is needed to initiate empiric therapy [4].

Nocardial brain abscess is life-threatening with high mortality, and as such empiric therapy along with proper surgical intervention, when indicated, is required. Complications are not only a direct result of the infection itself but can also occur because of the pharmacological and surgical interventions needed for treatment [3]. In the case described above, given the initial high suspicion of a brain tumor and the large size of the lesion (~3.5 cm), the patient immediately underwent a craniotomy. He was not initiated on empiric antimicrobial therapy straightaway given a low suspicion for an infectious etiology. After he was found to have an abscess, cultures were sent, and he was initiated on amikacin and Bactrim for a total of six weeks. He was later transitioned to ciprofloxacin to complete a full year of treatment. His hospital course was complicated by intraparenchymal hemorrhage as a result of the craniotomy. He then developed seizure activity initially due to the brain abscess and later as a consequence of the hemorrhagic foci. Fortunately, his mentation improved post-abscess resection, the seizures were controlled with anti-epileptic agents and the hemorrhage was limited and required no further intervention. He also developed pancytopenia relayed to trimethoprim/sulfamethoxazole and required supportive transfusions and hospital admission. This was resolved with the removal of the offending agent. At his one-year follow-up, the patient was reported to be mentally intact. He did have some persistent lower extremity muscle weakness, which was likely secondary to the multiple hospital admissions and deconditioning.

The guidelines for the management of nocardial brain abscess are lacking. Antimicrobial therapy, surgical interventions, or a combination of both have been proposed in the literature [3]. Multiple antibiotic agents and combinations have been suggested for treatment. The most common agents used include imipenem, meropenem, amikacin, quinolones, third-generation cephalosporins, linezolid, and trimethoprim/sulfamethoxazole [5,8]. Empiric coverage is usually with two or three agents with a combination of trimethoprim/sulfamethoxazole and imipenem with or without amikacin being the most preferred. Antibiotics should be tailored to culture once available. Intravenous therapy is recommended for six weeks, followed by oral therapy for six months to one year [3]. If the lesion is larger than 2.5 cm in diameter, then aspiration is recommended for decompression and confirmation of diagnosis [8]. Craniotomy and excision of the abscess should be performed if the abscess enlarges after two weeks of therapy or fails to shrink after four weeks of therapy [3].

## Conclusions

Although rare, nocardial brain abscess should always be considered within the differentials for immunocompromised patients presenting with neurological changes and concern for brain abscess on imaging, given its reported rapidly deteriorating clinical course. Empiric treatment can help prevent mortality and decrease morbidity. Given the rare occurrence of nocardial brain abscess, specific guidelines for the diagnosis and medical management are lacking. We hope this case will provide further reference.
